# Security bound of cheat sensitive quantum bit commitment

**DOI:** 10.1038/srep09398

**Published:** 2015-03-23

**Authors:** Guang Ping He

**Affiliations:** 1 School of Physics and Engineering, Sun Yat-sen University, Guangzhou 510275, China

## Abstract

Cheat sensitive quantum bit commitment (CSQBC) loosens the security requirement of quantum bit commitment (QBC), so that the existing impossibility proofs of unconditionally secure QBC can be evaded. But here we analyze the common features in all existing CSQBC protocols, and show that in any CSQBC having these features, the receiver can always learn a non-trivial amount of information on the sender's committed bit before it is unveiled, while his cheating can pass the security check with a probability not less than 50%. The sender's cheating is also studied. The optimal CSQBC protocols that can minimize the sum of the cheating probabilities of both parties are found to be trivial, as they are practically useless. We also discuss the possibility of building a fair protocol in which both parties can cheat with equal probabilities.

Quantum bit commitment (QBC) is a two-party cryptography including the following phases. In the commit phase, Alice (the sender of the commitment) decides the value of the bit *b* (*b* = 0 or 1) that she wants to commit, and sends Bob (the receiver of the commitment) a piece of evidence, e.g., some quantum states. Later, in the unveil phase, Alice announces the value of *b*, and Bob checks it with the evidence. The interval between the commit and unveil phases is sometimes called the holding phase. A QBC protocol is called unconditionally secure if any cheating can be detected with a probability arbitrarily close to 1. Here Alice's cheating means that she wants to change the value of *b* after the commit phase, while Bob's cheating means that he tries to learn *b* before the unveil phase.

QBC is an essential primitive for building quantum multi-party secure computations and other “post-cold-war era” multi-party cryptographic protocols^1^,^2^. Unfortunately, it is widely believed that unconditionally secure QBC is impossible^3^,^4^. This result, known as the Mayers-Lo-Chau (MLC) no-go theorem, was considered as putting a serious drawback on quantum cryptography.

To evade the problem, the concept “cheat sensitive quantum bit commitment (CSQBC)” was proposed^5^,^6^,^7^,^8^,^9^,^10^, where the probability for detecting the cheating does not need to be arbitrarily close to 1. Instead, it merely requires the probability to be nonzero. With this loosen security requirement, many insecure QBC protocols can be regarded as secure CSQBC. Therefore, at the first glance it seems that CSQBC will be very easy to achieve.

But intriguingly, here we will show that there still exists boundary for the security of a typical class of CSQBC. Especially, Bob can always feel free to measure the quantum states to learn *b*, while he stands at least 50% chances to escape Alice's detection.

## Results

### Common features of CSQBC

By checking the existing CSQBC protocols^5^,^6^,^7^,^8^,^9^,^10^, we find that they all share the following common features (note that the names Alice and Bob are used reversely in Refs. 7, 9, 10):

1. During the holding phase, the receiver Bob owns a quantum system Ψ encoding Alice's committed bit *b*. (Ψ can either be prepared by the sender Alice, or be prepared by Bob and sent to Alice, who returns it to Bob after performing some certain operations according to her choice of *b*. It also does not matter whether Alice prepared and kept another quantum system entangling with Ψ.)
2. Bob knows the definitions of 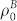 and 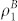 directly before the end of the commit phase. (That is, these definitions are either clearly stated by the protocol, or announced to Bob by Alice classically. Bob does not need to perform operations on any quantum system to gain knowledge of these definitions.) Here 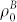 and 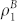 are the density matrices of Bob's Ψ corresponding to *b* = 0 and *b* = 1, respectively.
3. To detect Bob's cheating, at the unveil phase Alice can check whether the state of Ψ is intact. (It does not matter whether the entire Ψ or only a small part can be checked.)
4. To detect Alice's cheating, at the unveil phase Bob can learn a nontrivial amount of information on the value of *b* from Ψ, even without any help from Alice.

The last feature indicates that there exists at least one operation known to Bob, which can output a bit *b*′ when being applied on Ψ, and *b*′ = *b* should occur with a probability larger than 1/2. As a result, there must be 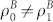. This is a main difference from the original QBC, where there is generally 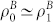 so that it can be unconditionally secure against dishonest-Bob.

The original purpose of CSQBC having these features is as follows. Alice's cheating strategy suggested in the MLC no-go theorem is based on the Hughston-Jozsa-Wootters (HJW) theorem^11^, which applies to the case 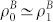. Therefore with feature (4), i.e., 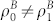, Alice's cheating becomes detectable so that the MLC no-go theorem can be evaded. On the other hand, if Bob takes advantages of 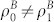 and performs measurements to discriminate the committed bit *b*, the quantum state will be disturbed. In this case, with feature (3) Bob's cheating will be detected with a certain probability when Alice asks him to return the quantum state and checks wether it remains undisturbed, so that the goal of CSQBC can be met.

But with a rigorous quantitative analysis on the probability of detecting Bob's cheating, we will find that it is always not sufficiently large when Bob applies some specific measurements. Therefore any CSQBC protocol having the above four features will be bounded by the security limit below.

### Notations and Bob's cheating strategy

According to Eq. (9.22) of Ref. 12, the trace distance 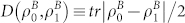 (where 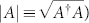) between 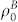 and 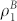 satisfies
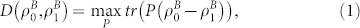
where the maximization is taken over all positive operators *P* ≤ *I*, with *I* being the identity operator. The above feature (2) of CSQBC guarantees that Bob knows how 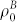 and 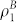 are defined. Thus he can find the positive projectors *P* = *P_m_* that maximizes 
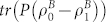
. If 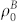 stands a higher probability to be projected successfully than 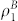 when applying *P_m_*, then we takes *P*_0_ ≡ *P_m_* and *P*_1_ ≡ *I* − *P_m_*. Otherwise we takes *P*_0_ ≡ *I* − *P_m_* and *P*_1_ ≡ *P_m_*. Feature (1) ensures that Bob owns the system Ψ encoding Alice's committed bit *b* during the holding phase. Therefore, by applying the positive operator-valued measure (POVM) 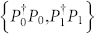 on Ψ, Bob can discriminate between 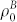 and 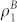 and learn Alice's committed *b* with the maximal probability allowed by 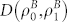.

To analyze rigorously the probability for Bob to escape Alice's detection with this POVM, let *H* be the global Hilbert space constructed by all possible states of Ψ (either *b* = 0 or 1). Since *P*_0_, *P*_1_ are positive projectors, there exists an orthonormal basis {|*e_i_*〉} of *H* (the following proof remains valid regardless whether {|*e_i_*〉} is known to Alice or Bob), in which *P*_0_, *P*_1_ can be expressed as
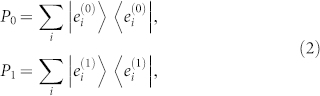
where 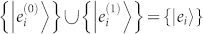.

Meanwhile, before Bob applying any measurement, the general form of the initial state of Ψ can always be written as
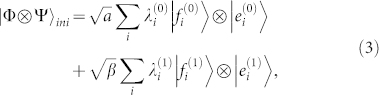
where 0 ≤ *α* ≤ 1, *β* = 1 − *α*, and 
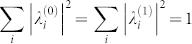
 (sum over all possible *i* within each corresponding subspace). The values of *α*, *β*, 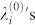 and 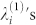 are chosen by Alice according to the value of her committed bit *b*. Here Φ is a quantum system that Alice may introduce and keep to herself, which entangles with Bob's Ψ. All 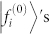 and 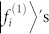 are the vectors describing the state of Φ, which are not required to be orthogonal to each other. In the case where Alice does not introduce such a system, we can simply set all 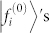 and 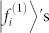 to be equal, so that Eq. (3) still applies.

### The security bound on Bob's cheating

As elaborated in the 1st subsection of Methods section, when dishonest-Bob applies the above POVM 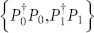 on Ψ, we find that the probability for Bob's cheating to pass Alice's detection successfully is
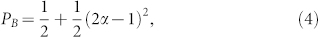
and the amount of mutual information he obtained is
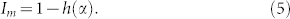
Here *h*(*α*) ≡ −*α* log_2_ *α* − (1 − *α*) log_2_(1 − *α*) is the binary entropy function.

**Figure 1:**
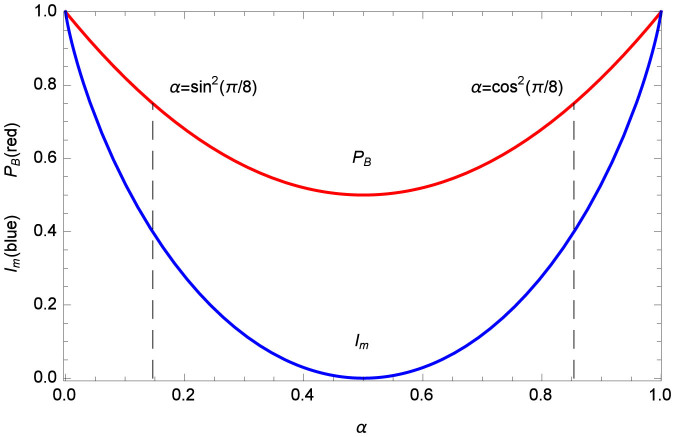
Bob's successful cheating probability *P_B_* (red line) and mutual information *I_m_* (blue line) on Alice's committed bit *b* as a function of *α* that Alice chooses for the initial state Eq.(3). The dash lines mark the values for the protocol in Ref. 5.

With Eqs. (4) and (5), we plot *P_B_* and *I_m_* as a function of *α* in FIG. 1. Since 0 ≤ *α* ≤ 1, FIG. 1 and Eq. (4) both gives
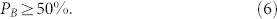
The minimum *P_B_* = 50% can be reached when Alice chooses *α* = 0.5. Thus we come to the conclusion that Bob can always learn Alice's committed *b* with the maximal probability allowed by the trace distance between 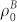 and 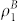, while his cheating stands at least 50% chance to escape Alice's detection.

It may look weird that FIG. 1 seems to indicate that the more amount of information that Bob obtains, the easier he can pass Alice's detection. But we must note that the amount of Bob's information is not chosen by himself. Instead, it is determined by the value of *α* that Alice chooses. That is, once Alice determines which state is used for encoding her committed bit, the maximum amount of information that Bob can obtain is also fixed.

On the other hand, the above result indicates that Alice should make *α* as close to 0.5 as possible, so that Bob's information and successful cheating probability can be minimized. However, note that she has to choose the initial state Eq. (3) within the range restricted by the protocol. Due to the feature (4) of CSQBC, the trace distance 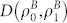 has to be nonzero, Therefore, generally *α* cannot be made very close to 0.5, as we will see in the examples below.

### Examples

In the CSQBC protocol in Ref. 5, Bob's system Ψ is a single qubit, whose state is either |0〉 or |−〉 (|1〉 or |+〉) when Alice commits *b* = 0 (*b* = 1). Here |0〉 and |1〉 are orthogonal to each other, 
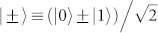
. So we have 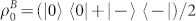 and 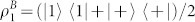. Define
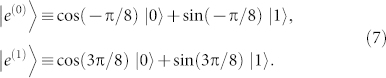
Then Bob's operation for maximally discriminating 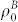 and 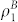 is to measure Ψ in the basis {|*e*^(0)^〉, |*e*^(1)^〉}, i.e., he applies the projector *P*_0_ = |*e*^(0)^〉 〈*e*^(0)^|. When the projection is successful (unsuccessful), he takes *b*′ = 0 (*b*′ = 1) as the decoded result. With this method, *b*′ will match Alice's actual committed bit *b* with the probability 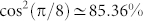. Meanwhile, Alice's four input states can be expanded in the {|*e*^(0)^〉, |*e*^(1)^〉} basis as
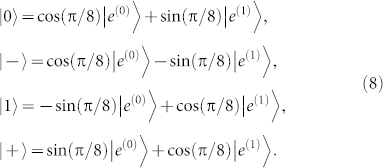
Comparing with Eq. (3), we can see that there is either *α* = cos^2^(*π*/8) or *α* = sin^2^(*π*/8). Substitute them into Eq. (4) will both yield *P_B_* = sin^4^(*π*/8) + cos^4^(*π*/8) = 75%. That is, in the CSQBC protocol in Ref. 5, Bob can learn Alice's committed bit with reliability 85.36% (i.e., his mutual information is 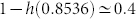 bit) before the unveil phase, while he can pass Alice's security check with probability 75%. This protocol is corresponding to the dash lines in our FIG. 1.

Another example can be found in Ref. 13, where we illustrated how our above cheating strategy applies on the CSQBC protocol in Ref. 9. This protocol looks more complicated than the one in Ref. 5, as the committed bit *b* is encoded with many qubits, instead of a single one. The authors of Ref. 9 merely analyzed the individual attack of the receiver (note that they used the names Alice and Bob reversely) where the qubits are measured one by one. Then it is concluded that the cheating can be detected with a probability arbitrarily close to 1. But as we shown above, instead of individual measurements, the dishonest receiver can apply a two-element POVM 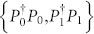 on the entire state encoding the committed bit. When this state consists of many qubits, each basis vector |*e_i_*〉 of the Hilbert space *H* is a multi-level state describing all qubits. Thus the projectors *P*_0_, *P*_1_ in Eq. (2) are actually collective measurements. The detailed form of *P*_0_, *P*_1_ is given in Eq. (2) of Ref. 13. As a result, it was further elaborated there that this collective measurement is as effective as individual measurements on learning the committed bit, while it causes much less disturbance on the multi-qubit state. Once again, the probability for the cheater to escape the detection was shown^13^ to be not less than 50%. With the increase of the qubit number *n*, this probability can even be arbitrarily close to 100%.

### Alice's cheating strategy

Alice's cheating strategy used in the MLC no-go theorem requires the condition 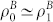, which no longer holds in CSQBC. Nevertheless, she can still apply the same strategy in CSQBC and try her luck. To give a detailed description of the strategy, first let us model the coding method in CSQBC more precisely. For generality, consider that in the protocol, besides Bob's system Ψ, there is another system *E*. Alice's different committed values of *b* is encoded with different states of the combined system *E* ⊗ Ψ. System *E* is kept at Alice's side during the commit and holding phases, and is required to be sent to Bob at the unveil phase to justify Alice's commitment. Let 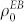 and 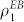 denote the density matrices of *E* ⊗ Ψ corresponding to *b* = 0 and *b* = 1, respectively. Note that in all existing CSQBC protocols^5^,^6^,^7^,^8^,^9^,^10^, there is no such a system *E*. But we include it here, so that the model can cover more protocols that may be proposed in the future.

In this scenario, Alice's cheating strategy is as follows. At the beginning of the protocol she introduces an ancillary system Φ which is a copy of *E* ⊗ Ψ. Since the fidelity 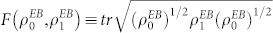 between 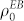 and 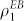 satisfies^12^
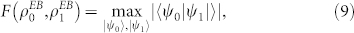
where the maximization is over all purifications |*φ*_0_〉 of 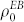 and |*φ*_1_〉 of 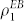 into Φ ⊗ *E* ⊗ Ψ, Alice finds the real and positive |*ψ*_0_〉, |*ψ*_1_〉 that reach the maximum, i.e.,
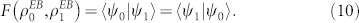
Then she prepares the initial state of Φ ⊗ *E* ⊗ Ψ as
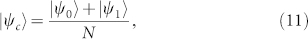
where the normalization constant
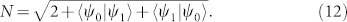
She uses this state to complete the rest of the commit protocol. With this method, the value of *b* is not determined during the commit phase.

In the unveil phase, Alice decides whether she wants to unveil *b* = 0 or *b* = 1. Then she simply uses |*ψ_c_*〉 as |*ψ_b_*〉 to complete the protocol. From the symmetry of |*φ*_0_〉 and |*φ*_1_〉 in Eq.(11), we can see that her successful cheating probabilities for *b* = 0 and *b* = 1 are both
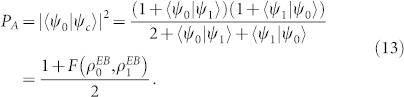
Therefore, in any specific CSQBC protocol, the Alice's exact cheating probability can be calculated once the definition of 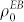, 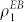 is known.

### The optimal protocols are trivial

Now we will try to find the CSQBC protocols which can optimally detect the cheating of both parties, i.e., minimizing the sum of Alice's and Bob's cheating probabilities.

Note that Eq. (4) depends on the specific value of *α* in the state Eq. (3) that Alice chooses in a single run of the protocol, while 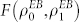 in Eq. (13) is the statistical result of all the legitimate states allowed by the protocol. Thus it is hard to compare Eq. (13) and Eq. (4) directly and give a general result without knowing the details on the composition of 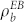 in a specific protocol.

Fortunately, in all existing CSQBC protocols^5^,^6^,^7^,^8^,^9^,^10^, there is no system *E*. The form of the states of Bob's system Ψ alone carries all the information of *b*. Thus the trace distance 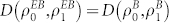. For any protocol of this kind (as well as protocols having system *E* but still satisfying 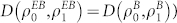), we can replace both *α* and 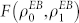 with 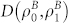, as elaborated in the 2nd subsection of Method, where we obtain
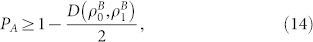
and
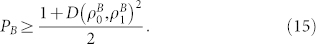


These two equations suggest that *P_A_* and *P_B_* cannot be minimized simultaneously in the same protocol, because reducing *P_A_* requires a higher 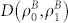, while it will result in a higher *P_B_* at the same time.

Moreover, we must note that the above *P_A_* and *P_B_* are obtained assuming that the actions of both parties in the protocol will always be checked. But this is impossible, because they share the same system Φ ⊗ *E* ⊗ Ψ. In the unveil phase, either Bob will measure *E* ⊗ Ψ to check Alice's action, or he is required to return Ψ to Alice who checks his action. These cannot be done simultaneously. Suppose that in a CSQBC protocol, Bob's action is checked with probability *ζ* (0 ≤ *ζ* ≤ 1), and Alice's action is checked with probability 1 − *ζ*. When one's action is not checked, he/she can cheat successfully with probability 1. Thus the cheating probabilities *P_A_* and *P_B_* should be replaced by
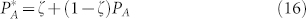
and
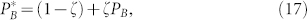
respectively. Combining them with Eqs. (14) and (15), we find
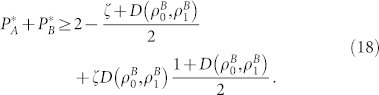
Since 0 ≤ *ζ* ≤ 1 and 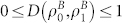, we find another security lower bound of CSQBC
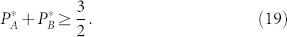


**Figure 2:**
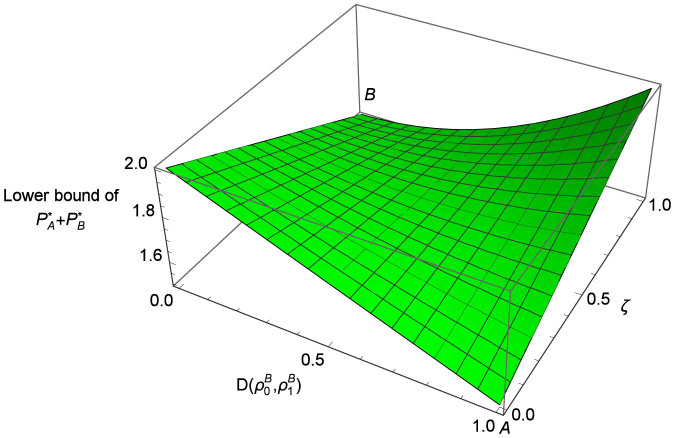
The lower bound of the sum of the cheating probabilities 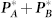 as a function of the trace distance 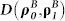 and the probability *ζ* with which Bob's action is checked. *A* and *B* denote the points that reach the minimum 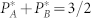.

To find the optimal protocol that can reach this bound, we plot the lower bound of 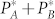 as a function of 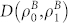 and *ζ* in FIG. 2 according to Eq. (18). It shows that there are two types of protocols that can both reach the minimum 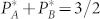, denoted as points *A* and *B* in FIG. 2, respectively, with the parameters (A) 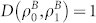, *ζ* = 0, and (B) 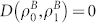, *ζ* = 1. Type (A) protocols mean that 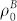 and 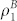 are orthogonal so that 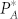 reaches its minimum 1/2. However, 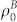 and 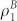 can be distinguished perfectly and Bob's action is never checked. Thus 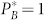, i.e., he can always learn Alice's committed *b* with reliability 1 and never get caught. In type (B) protocols, 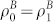 so that Bob learns nothing about *b*. But Alice's action is never checked so that she can unveil *b* as whatever she wants, with a successful cheating probability 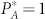. Therefore, we can see that these optimal protocols are all trivial as they are completely insecure against one of the parties. Thus they do not seem to have any practical usage.

### The fair protocol

Since the protocols that can minimize 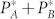 all look useless, let us consider the protocol satisfying 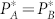 so that it is fair for both parties, and try to minimize 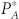, 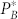 in this case. From Eq. (37) we can see that the inequality Eq. (15) can become equality when 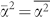, i.e., all the states allowed to be chosen in the protocol for committing the same *b* value should have the same *α* value. Also, note that the lowest bounds in Eqs. (14) and (18) cannot be reached by most 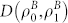, because these inequalities can become equalities if and only if 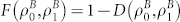, which requires 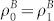. Therefore, only the above optimal protocols can reach these bound. For this reason, to calculate 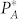 precisely in other protocols, we should use Eq. (13) instead of Eq. (14). To compute 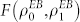 in Eq. (13), for simplicity we consider only the protocols in which there are
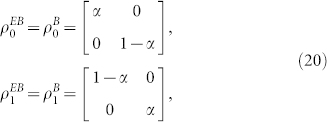
then
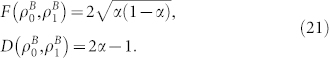
Combining them with Eqs. (13), (16), (17) and (15) (the latter becomes equality once we choose 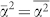), then by solving 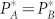 we yield
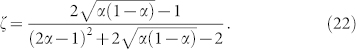
Any protocol satisfying this equation is fair for both parties. Now let us find the minimal value of 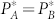. Substituting this *ζ* into Eq. (17), we obtain
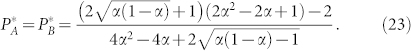
By solving 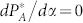, we find that the minimal cheating probabilities in such protocols are 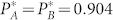, which can be obtained when 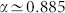, i.e., 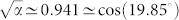. In this case 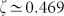.

A simple protocol having these parameters is: Alice sends Bob the state cos(19.85°) |0〉 ± sin(19.85°) |1〉 (sin(19.85°) |0〉 ± cos(19.85°) |1〉) if she wants to commit *b* = 0 (*b* = 1). In the unveil phase, with probability 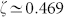 Bob returns the state and Alice checks whether it remains undisturbed, with probability 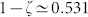 Bob measures the state and checks whether it agrees with the value of Alice unveiled *b*.

Nevertheless, there is the difficulty in finding a method for deciding which party will be checked in a single run of the protocol. Dishonest Alice (Bob) would like to decrease 1 − *ζ* (*ζ*) so that 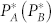 can be raised. Thus they do not trust each other and may not collaborate. The CSQBC protocol in Ref. 5 adopts a process called “the game” to handle this problem, which is very similar to quantum coin flipping (QCF) protocols^14^. However, Ishizaka^15^ showed that this process provides extra security loophole to Bob, so that there is a cheating strategy for him to learn *b* with reliability 61.79% (which is lower than what can be obtained with our cheating strategy, as calculated in the Examples section) while passing Alice's check with probability 100% (which is higher than that of our strategy). It was further shown in Ref. 16 that due to the inexistence of ideal black-boxed QCF, any CSQBC protocol based on biased QCF cannot be secure. Therefore, it remains unclear how to build a fair CSQBC protocol with 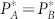 while minimizing 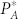 and 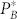.

## Discussion

In summary, we showed that any CSQBC protocol having the above four features is subjected to the security bound Eq. (6). Protocols satisfying 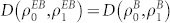 is further bounded by Eq. (19). Note that the insecurity of QCF-based CSQBC protocols (e.g., Refs. 5, 6) was already pinpointed out in Refs. 15, 16. But our proof also applies to the non-QCF-based ones.

Our result should not be simply considered as a generalization of the MLC no-go proof. Instead, it is a complement. This is because the MLC no-go proof applies to QBC protocol with 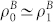. But as pointed out in Ref. 9, CSQBC does not need to satisfy this requirement so that it may evade the MLC theorem. On the contrary, our proof works for the case 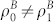, thus it fills the gap where the MLC proof left. Meanwhile, the MLC theorem concentrates on the cheating of Alice. It does not exclude the existence of protocols which is unconditionally secure against dishonest Bob only. On the other hand, our result shows that Bob can always cheat in CSQBC regardless Alice is honest or not.

It will be interesting to study whether there can be CSQBC protocols without the above four features. It seems that Kent's relativistic QBC^17^,^18^,^19^ and our recent proposals^20^,^21^ do not satisfy feature (1), while the protocol in Ref. 22 does not have feature (2), as elaborated in Ref. 23. However, these works are aimed to achieve the original QBC, instead of CSQBC. Also, Refs. 20,21,22,23 have not gained wide recognition yet. Thus it is still an open question whether it is possible to build non-relativistic CSQBC protocols which are not limited by the above security bounds, without relying on computational and experimental constraints.

## Methods

### Calculating Bob's cheating probability

Consider the POVM {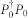, 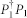} defined in Eq. (2). After Bob applies it on Ψ, there can be two outcomes.

(I) The projection outcome is *P*_0_. Then Bob takes *b*′ = 0 as his decoded result of Alice's committed bit *b*. With Eqs. (2) and (3) we yield
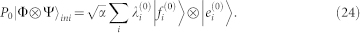
Thus this case will occurs with the probability
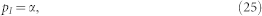
while the resultant state of Φ ⊗ Ψ is
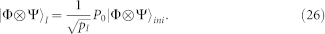


As described in feature (3) of CSQBC, at the unveil phase Alice may require Bob to return Ψ and check whether it remains intact in its initial state. The maximal probability for Alice to find out that Bob has already projected |Φ ⊗ Ψ〉*_ini_* into |Φ ⊗ Ψ〉*_I_* is bounded by
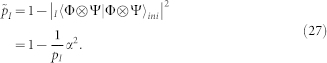
Thus the total probability for (case (I) occurred) *AND* (Alice failed to detect Bob's cheating) is
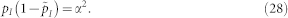


(II) The projection outcome is *P*_1_. Then Bob takes *b*′ = 1 as his decoded result of Alice's *b*. Now
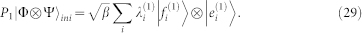
Obviously, this case will occurs with the probability
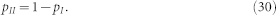
Meanwhile, the resultant state of Φ ⊗ Ψ in this case is
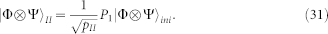


The maximal probability for Alice to find out that Bob has already projected |Φ ⊗ Ψ〉*_ini_* into |Φ ⊗ Ψ〉*_II_* is bounded by
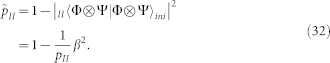
Thus the total probability for (case (II) occurred) *AND* (Alice failed to detect Bob's cheating) is
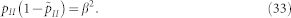


Taking both cases (I) and (II) into consideration, the overall probability for Bob's cheating to pass Alice's detection successfully is
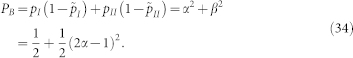


Meanwhile, since the projection outcome will either be *P*_0_ or *P*_1_ with the probabilities *p_I_* and *p_II_* = 1 − *p_I_*, respectively, Bob's *b*′ will match Alice's *b* with the probability *p_I_* or 1 − *p_I_* too. Note that *h*(1 − *p_I_*) = *h*(*p_I_*). Thus the amount of mutual information that Bob obtains with this POVM is
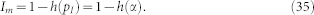


### Bounding the cheating probabilities with trace distance

Suppose that there are many states allowed to be chosen randomly for committing *b* = 0 in the protocol, each of which takes the form of Eq. (3), but with different values of the coefficients *α*, *β*, 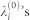 and 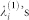. Bob applies the optimal POVM to decode *b*. Then Eq. (3) indicates that he can learn *b* correctly with probability 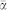, i.e., the average of *α*. Meanwhile, it is well-known that the maximal probability for discriminating two density matrices 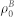, 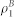 is 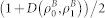. Therefore
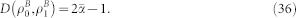
Since Eq. (4) shows that Bob's average cheating probability for these states is
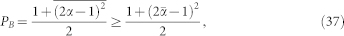
we have
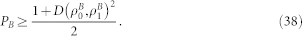
Similar discussion is also valid for the states for committing *b* = 1, except that *α* should be replace by *β* = 1 − *α*. But Eq. (38) remains the same because Eq. (4) satisfies *P_B_*(1 − *α*) = *P_B_*(*α*).

On the other hand, since^12^
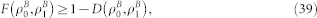
from Eq. (13) we yield
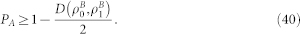


## References

[b1] Yao A. C. C. Security of quantum protocols against coherent measurements. In: Proc. 26th Symposium on the Theory of Computing. New York: ACM, pp. 67 (1995).

[b2] Kilian J. Founding crytpography on oblivious transfer. In: Proc. 1988 ACM Annual Symposium on Theory of Computing. New York: ACM, pp. 20 (1988).

[b3] Mayers D. Unconditionally secure quantum bit commitment is impossible. Phys. Rev. Lett. 78, 3414 (1997).

[b4] Lo H.-K. & Chau H. F. Is quantum bit commitment really possible? Phys. Rev. Lett. 78, 3410 (1997).

[b5] Hardy L. & Kent A. Cheat sensitive quantum bit commitment. Phys. Rev. Lett. 92, 157901 (2004). 15169319 10.1103/PhysRevLett.92.157901

[b6] Aharonov D., Ta-Shma A., Vazirani U. V. & Yao A. C. Quantum bit escrow. *arXiv:quant-ph/0004017v1*. In: Proc. 32nd Annual Symposium on Theory of Computing. New York: ACM, pp. 705 (2000).

[b7] Jakoby A., Liskiewicz M. & Madry A. Using quantum oblivious transfer to cheat sensitive quantum bit commitment. *arXiv:quant-ph/0605150v1* (2006).

[b8] Buhrman H., Christandl M., Hayden P., Lo H.-K. & Wehner S. Possibility, impossibility, and cheat sensitivity of quantum-bit string commitment. Phys. Rev. A 78, 022316 (2008). 10.1103/PhysRevLett.97.250501 17280334

[b9] Shimizu K., Fukasaka H., Tamaki K. & Imoto N. Cheat-sensitive commitment of a classical bit coded in a block of m × n round-trip qubits. Phys. Rev. A 84, 022308 (2011).

[b10] Li Y.-B., Wen Q.-Y., Li Z.-C., Qin S.-J. & Yang Y.-T. Cheat sensitive quantum bit commitment via pre- and post-selected quantum states. Quant. Inf. Process. 13, 141 (2014).

[b11] Hughston L. P., Jozsa R. & Wootters W. K. A complete classification of quantum ensembles having a given density matrix. Phys. Lett. A 183, 14 (1993).

[b12] Nielsen M. A. & Chuang I. L. in Quantum computation and quantum information, Ch. 9.2, 404–416 (Cambridge, 2000).

[b13] He G. P. Comment on “Cheat-sensitive commitment of a classical bit coded in a block of m × n round-trip qubits”. Phys. Rev. A 89, 056301 (2014).

[b14] Bennett C. H. & Brassard G. Quantum cryptography: public key distribution and coin tossing. In: Proceedings of the IEEE International Conference on Computers, Systems, and Signal Processing, 175 (IEEE Press, New York, 1984).

[b15] Ishizaka S. Is cheat sensitive quantum bit commitment really possible? *arXiv:quant-ph/0703099v3* (2007).

[b16] Ishizaka S. Dilemma that cannot be resolved by biased quantum coin flipping. Phys. Rev. Lett. 100, 070501 (2008). 18352530 10.1103/PhysRevLett.100.070501

[b17] Kent A. Unconditionally secure bit commitment. Phys. Rev. Lett. 83, 1447 (1999). 10.1103/PhysRevLett.109.130501 23030073

[b18] Kent A. Unconditionally secure bit commitment with flying qudits. New J. Phys. 13, 113015 (2011).

[b19] Kent A. Unconditionally secure bit commitment by transmitting measurement outcomes. Phys. Rev. Lett. 109, 130501 (2012). 23030073 10.1103/PhysRevLett.109.130501

[b20] He G. P. Quantum key distribution based on orthogonal states allows secure quantum bit commitment. J. Phys. A: Math. Theor. 44, 445305 (2011).

[b21] He G. P. Simplified quantum bit commitment using single photon nonlocality. Quantum Inf. Process. 13, 2195 (2014).

[b22] He G. P. Secure quantum bit commitment against empty promises. Phys. Rev. A 74, 022332 (2006).

[b23] He G. P. Secure quantum bit commitment against empty promises. II. The density matrix. *arXiv:1307.7318* (2013).

